# Demographic and socioeconomic inequalities in oral healthcare utilisation in Malaysia: evidence from a national survey

**DOI:** 10.1186/s12903-020-01388-w

**Published:** 2021-01-19

**Authors:** Yeung R’ong Tan, Ee Hong Tan, Suhana Jawahir, Ainul Nadziha Mohd Hanafiah, Muhammad Hafiz Mohd Yunos

**Affiliations:** 1 Institute for Health Systems Research, National Institutes of Health, Ministry of Health Malaysia, Blok B2, Kompleks NIH, No. 1, Jalan Setia Murni U13/52, Seksyen U13 Setia Alam, 40170 Shah Alam, Selangor Malaysia; 2 Tengkawang Dental Clinic, Terengganu Health Department, Ministry of Health Malaysia, 21700 Hulu Terengganu, Terengganu, Malaysia

**Keywords:** Oral health, Dental care, Adult, Socioeconomic factors, Healthcare inequalities, Malaysia

## Abstract

**Background:**

Throughout the years, oral healthcare utilisation in Malaysia has been low despite various efforts by the Ministry of Health Malaysia for improvement. This study aimed to determine the prevalence of oral healthcare utilisation and identify factors associated with oral healthcare utilisation among adults in Malaysia.

**Methods:**

Secondary data analysis of adults aged 18 years and over from the National Health and Morbidity Survey 2019 was conducted in this study. Characteristics of respondents and those who utilised oral healthcare were described using complex sample descriptive statistics. Logistic regression analysis was performed to examine the association between the dependent and independent variables. Dependent variable was oral healthcare utilisation in the last 12 months. Independent variables were demographic and socioeconomic factors (predisposing, enabling and need characteristics) based on Andersen’s Behavioural Model.

**Results:**

A total of 11,308 respondents, estimated to represent 21.7 million adults aged 18 years and over in Malaysia were included in the analysis. Prevalence of oral healthcare utilisation in the last 12 months was 13.2%. Demographic factors of sex, age, marital status, and socioeconomic factors of education level and occupation as well as health belief such as medical check-up were significantly related to oral healthcare utilisation. Enabling factor of household income quintile had significant association with oral healthcare utilisation. Inequalities were observed; females (OR = 1.57, 95% CI = 1.25, 1.96), younger adults (OR = 1.64, 95% CI = 1.15, 2.33), those who were married (OR = 1.65, 95% CI = 1.23, 2.22), those with higher education (OR = 2.21, 95% CI = 1.23, 3.99), those who had medical check-up in the last 12 months (OR = 1.86, 95% CI = 1.53, 2.25) and those with higher income (OR = 1.43, 95% CI = 1.04, 1.96) were more likely to utilise oral healthcare.

**Conclusion:**

Understanding factors associated with utilisation of oral healthcare could help in formulating effective interventions to improve oral healthcare utilisation. Demographic and socioeconomic factors are strong determinants of oral healthcare utilisation in Malaysia. Appropriate interventions to strengthen the existing programmes aimed to promote regular and timely oral health check-ups are needed to improve oral healthcare utilisation.

## Background

Oral diseases share common risk factors with non-communicable diseases [1], affecting people throughout their lifetime and pose health burden for many countries. Untreated dental caries remain the most common health condition, while severe periodontal disease affects almost 10% of the global population, often resulting in tooth loss [2]. According to the National Oral Health Survey of Adults 2010, 88.9% of adults in Malaysia had dental caries which included treated and untreated dental caries, and 94% had periodontal conditions [3]. Assessment of treatment needs found that 54.1% of adults required treatment for dental caries and 87.2% required periodontal treatment [3]. The same study also indicated that almost everyone (98.3%) needed oral healthcare in the past year but less than one-third of adults utilised oral healthcare services in the past one year [3].

Most oral diseases are preventable and can be treated in their early stages [4]. Regular oral healthcare utilisation is important for prevention and early detection of oral diseases to maintain good general well-being. Thomson et al. [5] indicated better self-reported oral health, less tooth loss and dental caries, and cleaner teeth in routine attendees than problem-oriented attendees of oral healthcare.

Oral healthcare in Malaysia is dichotomous, with a public sector which provides universal healthcare through the Ministry of Health Malaysia and a co-existing private sector. Malaysia is one of the early achievers of universal health coverage [6] through the provision of comprehensive public healthcare services funded by general taxation. Performance monitoring of the Sustainable Development Goals and Universal Health Coverage (UHC) shows Malaysia scores a UHC service coverage index of 73 out of 100, which is higher than the average of the middle-income countries (64.9) [7]. This achievement is also higher than neighbouring ASEAN countries, except Singapore, Brunei, Thailand, and Vietnam (with scores ranged from 75 to 86) [7]. In Malaysia, oral healthcare is largely subsidised in the public sector where basic oral healthcare is accessible to all citizens with a nominal fee or for free, while the private sector renders services to all through fee-for-service. Preschool children, schoolchildren seventeen years and below, antenatal mothers, civil servants and the physically, mentally, and economically disadvantaged groups are entitled to free basic oral healthcare [8]. In the purview of oral healthcare for children, many lie under the public sector through the Incremental School Oral Health Programme, while adult oral healthcare lies under the public and private sectors [8]. There is no national health insurance scheme in place but third party payment schemes are available, albeit very few [8].

Many studies worldwide have addressed the frequency and determinants of oral healthcare utilisation [9–12]. Age, sex, urbanity, education level, income level, employment, and health status among others have been associated with oral healthcare utilisation in many studies around the world [9–12]. However, published research on associated factors of oral healthcare utilisation in Malaysia is scarce. Demographics, socioeconomic backgrounds, and beliefs differ in different parts of the world which influences oral healthcare utilisation differently [13–15]. Understanding the associated factors of oral healthcare utilisation helps to fill knowledge gaps regarding demographic and socioeconomic inequalities which are essential for health system strengthening [16] and better planning of oral healthcare services.

Many theoretical models have attempted healthcare utilisation explanation; Dutton Model [17], Evans and Stoddart Model [18], Health Belief Model [19], Grossman Model of Health Demand [20], and Andersen’s Behavioural Model [21]. In our study, we adopted the Andersen’s Behavioural Model which includes predisposing factors such as demographic profile and socioeconomic status, as well as enabling and need factors at the individual level to conceptualise factors that influence access and utilisation. Besides its ease of implementation, Andersen’s Behavioural Model was chosen due to its popularity in modelling studies involving healthcare utilisation and accessibility [22].

In this study, we aimed to determine the prevalence of oral healthcare utilisation and use Andersen’s Behavioural Model to identify the associated factors of oral healthcare utilisation by adults in Malaysia.

## Methods

### Study design and sampling

A secondary data analysis was performed using subset of adults aged 18 years and over from the National Health and Morbidity Survey (NHMS) 2019.

### Source of data

NHMS 2019 was a cross-sectional, national household survey that targeted all non-institutionalised population who were residing in Malaysia for at least two weeks prior to data collection. Two-stage proportionate to size cluster sampling design was used to ensure national representativeness. All thirteen states and three Federal Territories were included, and stratification was performed by states and urban or rural localities. Primary stratum was states and Federal Territories, while secondary stratum was urban and rural strata formed within the primary stratum. First stage sampling involved random selection of all Enumeration Blocks (EBs) in Malaysia through probability proportional to size sampling technique. A total of 463 EBs were selected as primary sampling units, 350 EBs from the urban areas and 113 EBs from the rural areas. Second stage sampling involved random selection of Living Quarters (LQs) from the selected EBs. Fourteen LQs were randomly selected from each selected EB. Department of Statistics Malaysia performed the random selection of EBs and LQs. All households within the selected LQs and all eligible respondents in the households were included in the study. Of the 5,791 eligible LQs, a total of 5,365 LQs were successfully interviewed giving a LQ response rate of 92.6%. Respondents eligible for the survey were 18,546. A total of 16,688 respondents were successfully interviewed, giving an individual response rate of 90.0%. The overall response rate for this community-based survey is therefore 83.4%. A detailed methodology and sampling design of the survey is described in the NHMS 2019 official report [23].

### Data collection

Data collection was conducted from July to October 2019 by trained data enumerators via face-to-face interview using bilingual (Malay and English), structured and validated questionnaire [24]. The questionnaire was programmed into an application and uploaded onto tablets as data collection tools. Data collected were stored and backed-up in Secure Digital (SD) cards, and subsequently uploaded to a central system after quality check. Eligible residents from the selected households were invited to participate in the survey. Vacant or closed houses during the first visit were revisited at least three times to ensure required sample size was achieved. Throughout the conduct of the study, the tenets of the Declaration of Helsinki was followed. Written informed consent was obtained from all participants prior to the interviews. The Medical Research and Ethics Committee (MREC), Ministry of Health Malaysia granted the permission to carry out the National Health and Morbidity Survey 2019 (NMRR-18-3085-44207).

### Data analysis

Only respondents with complete data on all variables were included in the analysis, as the proportion of missing data was 3.14% (n = 366). Complete case analysis may be used as primary analysis if the missing data proportion is below 5% [25]. Analysis of all respondents including those with missing data in any variable included in the study was conducted beforehand, and produced the same results.

#### Oral healthcare utilisation

Oral healthcare utilisation (dependent variable) refers to oral healthcare utilisation by the respondents in the last 12 months prior to the interview. Oral healthcare utilisation was then assessed in relation to the potential determinants (independent variables).

#### Potential determinants

Predisposing factors include demographic profiles, socioeconomic status, and health belief of the respondents. Variables on the demographic profiles included sex, age, ethnicity, citizenship, and marital status. For socioeconomic status, education level, occupation, and working status were included. Utilisation of medical check-up were included under health belief where respondents were asked to answer “yes” or “no” to the question “In the last 12 months, did you go for medical check-up such as blood/urine tests/x-ray?”.

Enabling factors include household income quintile and urbanity (rural–urban location). Urbanity was included as enabling factor [22] because urban areas have higher density of oral healthcare practitioners and oral healthcare facilities [8].

For need factor, self-rated health status was included, and respondents were asked to answer using a five-point scale (excellent, good, fair, poor, very poor) to the question “How would you rate your health status?”. The responses were then grouped into three categories: (1) good to excellent health; (2) fair health; and (3) poor to very poor health, during data analysis.

Descriptive statistics were used to describe characteristics of respondents and those who utilised oral healthcare. Pearson’s Chi-square tests with 95% confidence intervals (CI) were used to compare the characteristics of respondents who used oral healthcare. Statistical significance was set at 0.05.

#### Logistics analysis

Univariate analysis was performed to examine the association between independent variables (predisposing, enabling and need factors), and oral healthcare utilisation. Crude odds ratios (OR) were used to estimate strength of association between independent and dependent variables. Variables with *p* value < 0.25 [26] in univariate analysis were included in the multivariate regression analysis. Multivariate logistic regression model was fitted to determine the associated factors of oral healthcare utilisation. Adjusted OR with 95% confidence interval were determined. A *p*-value of < 0.05 was considered statistically significant. The goodness of fit model was tested using Hosmer–Lemeshow statistics, and *p*-value > 0.05 was considered as good fit.

All analyses used complex sample descriptive statistics to account for sample weightage and study design properties. The weight used for estimation was based on the products of the inverse of the probability of sampling, the non-response adjustment factor, and a post-stratification adjustment by age, gender, and ethnicity. Statistical analysis was performed using STATA 14 (Stata Corporation, College Station, TX).

## Results

A total of 11,308 respondents, estimated to represent 21.7 million adults aged 18 years and over in Malaysia were included in the analysis. Table 1 summarises the sociodemographic characteristics of the respondents. Females made up 50.1% of the respondents and those aged 18–34 years old were 44.5% of the respondents. 51.4% of the respondents were Malay, 88.5% were Malaysian, and 63.1% were married. 48.8% of respondents reported their highest education level to be up to secondary education. Private employees made up 37.7% of the respondents and 62.1% were working. About one-third (33.1%) of the respondents had medical check-up. The 20% poorest made up 20.8% of the respondents. Majority of the respondents were from the urban locality (75.4%) and had self-rated good to excellent health (79.1%).

**Table 1:** Characteristics of respondents (N = 11,308)

Characteristics	Count, n (Unweighted)	Estimated population, N (Weighted)	Percentage (%)
*Predisposing factors*			
*Demographic profile*			
Sex			
Male	5345	10,808,937	49.9
Female	5963	10,844,610	50.1
Age group (years)			
18–34	3608	9,637,542	44.5
35–44	2246	4,187,976	19.3
45–59	3025	4,625,530	21.4
60 +	2429	3,202,499	14.8
Ethnicity			
Malay*	7409	11,122,297	51.4
Chinese	1429	4,630,824	21.4
Indian	727	1,323,426	6.1
Other Bumiputera	1114	2,266,043	10.5
Others	629	2,310,957	10.7
Citizenship			
Malaysian	10,603	19,162,080	88.5
Non-Malaysian	705	2,491,466	11.5
Marital status			
Single	2397	6,277,951	29.0
Married	7697	13,652,757	63.1
Widow(er)/Divorcee	1214	1,722,839	8.0
*Socio-economic status*			
Education level			
No formal education	666	1,191,602	5.5
Primary education	2492	4,315,337	19.9
Secondary education	5390	10,560,355	48.8
Tertiary education	2760	5,586,253	25.8
Occupation			
Government employee	1176	1,510,221	7.0
Private employee	3299	8,155,526	37.7
Self-employed	2132	3,851,320	17.8
Unpaid worker/Housewife	2064	3,705,499	17.1
Retiree	522	735,685	3.4
Student	312	863,511	4.0
Not economically active^†^	1803	2,831,784	13.1
Working status			
Not working	4742	8,213,140	37.9
Working	6566	13,440,407	62.1
*Health belief*			
Medical check-up			
Yes	4379	7,171,053	33.1
No	6929	14,482,494	66.9
*Enabling factors*			
Household income quintile			
Q1 (20% poorest)	2449	4,507,679	20.8
Q2	2230	4,281,591	19.8
Q3	2202	4,411,873	20.4
Q4	2247	4,234,963	19.6
Q5 (20% richest)	2180	4,217,440	19.5
Urbanity			
Urban	6772	16,335,491	75.4
Rural	4536	5,318,055	24.6
*Need factor*			
Self-rated health status			
Good to excellent health	8532	17,120,580	79.1
Fair health	2502	4,084,033	18.9
Poor to very poor health	274	448,933	2.1

*Q* quintile

^*^Includes Orang Asli

^**†**^Unemployed, health problem, old age

Prevalence of oral healthcare utilisation among adults aged 18 years and over in Malaysia in the 12 months preceding the study was 13.2% (95% CI = 12.0%, 14.5%). Comparison of characteristics of those who utilised oral healthcare is summarised in Table 2. Those who utilised had similar characteristics except for sex, age, marital status, education level, occupation, utilisation of medical check-up, and household income quintile.

**Table 2:** Characteristics of adults aged 18 years and over who utilised oral healthcare (N = 1596)

Characteristics	Count, n (Unweighted)	Estimated population, N (Weighted)	Prevalence, % (95% CI)	*p* value
Malaysia	1596	2,848,576	13.2 (12.0–14.5)	–
*Predisposing factors*				
*Demographic profile*				
Sex				
Male	574	1,136,872	10.5 (8.9–12.4)	< 0.001
Female	1022	1,711,703	15.8 (14.3–17.4)	
Age group (years)				
18–34	595	1,377,064	14.3 (12.3–16.5)	< 0.001
35–44	376	571,705	13.7 (11.7–15.9)	
45–59	420	637,588	13.8 (12.0–15.8)	
60 +	205	262,219	8.2 (6.7–10.0)	
Ethnicity				
Malay*	1101	1,592,232	14.3 (13.0–15.7)	0.093
Chinese	172	586,266	12.7 (9.8–16.2)	
Indian	109	203,961	15.4 (11.5–20.3)	
Other Bumiputera	170	313,197	13.8 (10.9–17.4)	
Others	44	152,921	6.6 (2.8–14.7)	
Citizenship				
Malaysian	1545	2,661,177	13.9 (12.7–15.1)	0.080
Non-Malaysian	51	187,399	7.5 (3.6–15.0)	
Marital status				
Single	346	815,440	13.0 (11.0–15.3)	0.001
Married	1138	1,906,892	14.0 (12.5–15.6)	
Widow(er)/Divorcee	112	126,244	7.3 (5.8–9.2)	
*Socioeconomic status*				
Education level				
No formal education	50	89,293	7.5 (5.0–11.1)	< 0.001
Primary education	193	282,622	6.5 (5.2–8.3)	
Secondary education	732	1,316,176	12.5 (10.9–14.2)	
Tertiary education	621	1,160,485	20.8 (18.2–23.6)	
Occupation				
Government employee	280	326,851	21.6 (18.2–25.5)	< 0.001
Private employee	453	1,046,103	12.8 (10.7–15.3)	
Self-employed	226	395,824	10.3 (8.4–12.5)	
Unpaid worker/Housewife	318	528,136	14.3 (12.3–16.5)	
Retiree	80	94,921	12.9 (9.2–17.8)	
Student	79	181,488	21.0 (14.7–29.1)	
Not economically active^**†**^	160	275,252	9.7 (7.7–12.3)	
Working status				
Not working	647	1,091,949	13.3 (11.8–15.0)	0.826
Working	949	1,756,627	13.1 (11.6–14.7)	
*Health belief*				
Medical check-up				
Yes	812	1,268,343	17.7 (15.9–19.6)	< 0.001
No	784	1,580,233	10.9 (9.5–12.5)	
*Enabling factors*				
Household income quintile				
Q1 (20% poorest)	246	467,068	10.4 (8.6–12.4)	< 0.001
Q2	261	464,069	10.8 (8.3–14.1)	
Q3	280	464,103	10.5 (8.8–12.6)	
Q4	343	597,262	14.1 (12.1–16.4)	
Q5 (20% richest)	466	856,073	20.3 (17.4–23.6)	
Urbanity				
Urban	1074	2,221,183	13.6 (12.1–15.2)	0.159
Rural	522	627,393	11.8 (10.0–13.9)	
*Need factor*				
Self-rated health status				
Good to excellent health	1190	2,234,735	13.1 (11.7–14.6)	0.730
Fair health	377	561,916	13.8 (11.8–15.9)	
Poor to very poor health	29	51,925	11.6 (7.0–18.6)	

*CI* confidence interval, *Q* quintile

^*^Includes Orang Asli

^**†**^Unemployed, health problem, old age

Table 3 displays the results of the logistic regression analysis of oral healthcare utilisation, with crude and adjusted odds ratios, and their confidence intervals and p-values.

**Table 3:** Logistics regression analysis of oral healthcare utilisation among adults aged 18 years and over

Variables	Crude OR (95% CI)	*p* value	Adjusted OR (95% CI)	*p* value
Sex				
Female	1.59 (1.29–1.97)	< 0.001	1.57 (1.25–1.96)	< 0.001
Male	*Ref*	*–*	*Ref*	–
Age group (years)				
18–34	1.87 (1.43–2.44)	< 0.001	1.64 (1.15–2.33)	0.007
35–44	1.77 (1.34–2.35)		1.42 (0.98–2.07)	0.064
45–59	1.79 (1.39–2.32)		1.44 (1.03–2.00)	0.031
60 +	*Ref*	–	*Ref*	–
Ethnicity				
Malay*	1.15 (0.85–1.57)	0.313		–
Indian	1.26 (0.81–1.94)			–
Other Bumiputera	1.11 (0.73–1.67)			–
Others	0.49 (0.19–1.24)			–
Chinese	*Ref*	–		–
Citizenship				
Malaysian	1.98 (0.91–4.33)	< 0.001	1.30 (0.59–2.88)	0.513
Non–Malaysian	*Ref*	–	*Ref*	–
Marital status				
Single	1.89 (1.41–2.53)	< 0.001	1.33 (0.88–2.02)	0.175
Married	2.05 (1.56–2.70)		1.65 (1.23–2.22)	0.001
Widow(er)/Divorcee	*Ref*	–	*Ref*	–
Education level				
No formal education	*Ref*	–	*Ref*	–
Primary education	0.87 (0.53–1.40)	< 0.001	0.83 (0.51–1.38)	0.477
Secondary education	1.76 (1.10–2.80)		1.42 (0.78–2.58)	0.246
Tertiary education	3.24 (2.04–5.14)		2.21 (1.23–3.99)	0.008
Occupation				
Government employee	2.57 (1.83–3.60)	< 0.001	1.06 (0.72–1.56)	0.774
Private employee	1.37 (0.98–1.90)		0.96 (0.68–1.34)	0.796
Self–employed	1.06 (0.77–1.48)		0.87 (0.60–1.24)	0.439
Unpaid worker/Housewife	1.54 (1.14–2.10)		0.98 (0.70–1.38)	0.922
Retiree	1.38 (0.90–2.10)		1.14 (0.69–1.86)	0.611
Student	2.47 (1.51–4.05)		1.52 (0.88–2.60)	0.130
Not economically active^†^	*Ref*	–	*Ref*	–
Working status				
Working	0.98 (0.82–1.17)	0.826		–
Not working	*Ref*	–		–
Medical check–up				
Yes	1.75 (1.46–2.11)	< 0.001	1.86 (1.53–2.25)	< 0.001
No	*Ref*	–	*Ref*	–
Household income quintile				
Q1 (20% poorest)	*Ref*	–	*Ref*	–
Q2	1.05 (0.74–1.49)	< 0.001	1.01 (0.72–1.41)	0.963
Q3	1.02 (0.78–1.33)		0.86 (0.65–1.16)	0.330
Q4	1.42 (1.11–1.81)		1.12 (0.85–1.46)	0.420
Q5 (20% richest)	2.20 (1.67–2.91)		1.43 (1.04–1.96)	0.026
Urbanity				
Urban	1.18 (0.94–1.48)	0.159	0.96 (0.77–1.20)	0.728
Rural	*Ref*	–	*Ref*	–
Self-rated health status				
Good to excellent health	1.15 (0.65–2.03)	0.708		–
Fair health	1.22 (0.69–2.16)			–
Poor to very poor health	*Ref*	–		–

*CI* confidence Interval, *OR* odds ratio, *ref* reference category, *Q* quintile

^*^Includes Orang Asli

^**†**^Unemployed, health problem, old age

Goodness of fit for final model: Hosmer–Lemeshow statistic:0.483

### Oral healthcare utilisation in the last 12 months

In the univariate analysis, variables with highly significant association with greater likelihood of using oral healthcare in the last 12 months were sex, age, citizenship, marital status, education level, occupation, utilisation of medical check-up in the last 12 months, and household income quintile (*p* < 0.001). The *p* value for urbanity was 0.159. All variables were included in the final model of multivariate analysis except ethnicity, working status, and self-rated health status (*p* values > 0.25 in the univariate analysis).

For predisposing factors, after controlling for all other variables in the model, the likelihood for oral healthcare utilisation in the last 12 months were higher among females (OR = 1.57, 95% CI = 1.25, 1.96). Younger adults had higher odds of oral healthcare utilisation compared to elderly aged 60 years and older. Those aged 18–34 years were 1.64 times more likely to use oral healthcare in the last 12 months compared to those aged 60 years and older. Married population had higher odds of oral healthcare utilisation (OR = 1.65, 95% CI = 1.23, 2.22) compared to widow(er)/divorcee. Adults with tertiary education had higher odds of oral healthcare utilisation in the last 12 months (OR = 2.21, 95% CI = 1.23, 3.99) compared to those with no formal education. Those with medical check-up in the last 12 months had higher odds of oral healthcare utilisation. For enabling factor, the richest 20% (Q5) were 1.43 times more likely to use oral healthcare compared to the poorest 20%. In the final model, status of citizenship, occupation, and urbanity were not significantly associated with oral healthcare utilisation. The *p *value for this model was > 0.05; this model is a good fit.

## Discussion

This study aimed to determine the prevalence of oral healthcare utilisation and used Andersen’s Behavioural Model to identify associated factors of oral healthcare utilisation by adults in Malaysia. Oral healthcare utilisation in the last 12 months by adults in Malaysia was 13.2%. Inequalities were observed; females, married individuals, younger adults, those with higher education, those who had medical check-up in the last 12 months, and those with higher income were more likely to utilise oral healthcare.

Oral healthcare utilisation in Malaysia is still low, considering the efforts done by the Ministry of Health Malaysia to encourage participation and utilisation of oral healthcare services. Every year, various oral health promotion activities such as community outreach programmes which offers free oral examination and awareness talk to target groups were conducted by the Ministry of Health Malaysia to increase oral health awareness and encourage oral healthcare utilisation among the public. However, participation is often low [27] which prompts a need for future studies to explore the reasons for the lack of enthusiasm among the public towards programmes conducted by the Ministry of Health Malaysia. Across 27 Organisation for Economic Co-operation and Development (OECD) countries, around 63% of individuals reported oral healthcare utilisation in the past year [28], which was higher compared to Malaysia. Compared to neighbouring countries like Thailand [range of 6.63% (aged 60 years and over)—8.81% (aged 15–24 years)] [29] and Indonesia [regular users: 1.2% (aged 15 years and over)] [10], Malaysia fared better in oral healthcare utilisation. However, Malaysia’s oral healthcare utilisation was low when compared to middle income countries like Brazil [less than one year since last dental visit: 44.4% (aged 18 years and over)] [30]. Nonetheless, these data are not directly comparable due to differences in methods and variable measured. Low oral healthcare utilisation may be associated with low perceived need for oral healthcare [31]. In 2010, adults in Malaysia who never sought oral healthcare stated not having any oral healthcare problem as the most common reason for non-utilisation of oral healthcare [3]. In 2015, 73.4% of the population in Malaysia experienced oral health problem(s) but did not seek oral healthcare, with the majority (46.1%) practising self-medication [32].

The NOHSA 2010 indicated that 27.4% of the adult population aged 15 years and above utilised oral healthcare in the past one year, a finding much higher than our study. A possible explanation could be the fact that the NOHSA 2010 was conducted by interviewers who were oral healthcare professionals while our study interview was conducted by research assistants who were not oral healthcare professionals [3]. Study have shown interviewer effect on respondents during data collection in public health surveys which tend to introduce a bias where the respondent reports the desired answer to escape negative consequences or out of fear [33, 34]. The NOHSA 2010 also included respondents aged 15 to 17 years old compared to our study which included only respondents aged 18 years and above [3]. The incremental school oral health programme in Malaysia covers students up to the age of 17 years old, which could explain the higher oral healthcare utilisation rate in NOHSA 2010 compared to our study [8].

Published literature suggests an inverse relationship between regular oral healthcare utilisation and increasing age [35]. In this study, working age adults (18–59 years) were more likely to utilise oral healthcare than the elderly (≥ 60 years). Lo et al. [36] found oral healthcare utilisation of preventive purpose in young adults (35 to 44 years) and curative purpose in the elderly (65 to 75 years). Harford et al. [37] indicated a high proportion of adults (25–44 years) going for oral health check-up. Oral Health Care Programme for the Elderly was introduced by the Ministry of Health Malaysia in 1983 as an initiative to promote oral health among the elderly [38]. Its implementation was confined within residential institutions, while a revised guideline in 2002 included national oral health goals such as domiciliary oral healthcare services, facilities and delivery systems improvement, elderly care training for oral health professionals and caregivers, and multi-sector collaborations to promote oral health for the elderly [38]. Oral healthcare utilisation among the elderly was still low despite the various efforts; 14.7% of Malaysians aged 60 years and over had never seen a dentist, and 86.1% did not use oral healthcare during the previous year [3]. Having a problem (65.2%), completing treatment (18.6%), and being sent a reminder (1.5%) was the main reason for their last oral healthcare utilisation [3]. The same study indicated that less than 10% of adults in Malaysia reported the need for timely oral healthcare utilisation [3]. Barriers to elderly oral healthcare utilisation were not specifically reported, with around 24.8% expressing some fear of oral healthcare utilisation [3].

This study showed that higher educated groups utilise oral healthcare services more than the less educated, a finding consistent with other study [13]. Education may be correlated with higher health consciousness which stimulates preventive behaviour such as regular oral healthcare utilisation [39]. According to findings from NHMS 2019, only 29.9% of population with oral health problems within two weeks prior to the interview perceived the need to seek treatment from a healthcare practitioner [23]. The main reasons for not seeking care from a healthcare practitioner when they had oral health problems were not being sick enough to necessitate treatment, work commitment, and self-medication [23]. These findings indicate that there is still a lack of awareness among the population in Malaysia regarding the importance of prevention in oral health. A national population study in Malaysia conducted in year 2019 showed sufficient health literacy level in only 40.7% of the population, followed by limited health literacy level in 35.0% and excellent health literacy level in 24.3% of the population [40]. However, data from the World Bank in 2018 shows that adults in Malaysia had literacy rate of 94.85% which is relatively high compared to some neighbouring country such as Thailand which had literacy rate of 93.77% in the same year [41]. This prompts the need to explore other factors such as local culture, beliefs, and taboos which could have contributed to the lack of awareness on the importance of prevention in oral health, leading to low utilisation of oral health services.

The higher odds of oral healthcare utilisation among population with medical check-up in this study could be attributed to the antenatal programme [8], as antenatal check-up was considered a form of medical check-up in NHMS 2019. Oral health is integral to general health. Having a health condition would necessitate a medical check-up and those with long standing health condition have been associated with higher utilisation of oral healthcare services [42]. Additional knowledge on oral health among other healthcare groups would ensure patient’s oral health needs are addressed and appropriate referral to the dentist is made during routine medical check-up. For instance, individuals with risk factor for oral health problem such as non-communicable diseases and smoking should be referred to the dentist whenever possible [2]. As such, medical, nursing, and allied-health programmes in institutions of higher learning could incorporate inter-professional and multidisciplinary oral health education as part of their curriculum to enable identification of oral health conditions and make appropriate referrals for oral healthcare, in preparation for future healthcare teams to manage people holistically in their general health as well as oral health [4, 43].

In our study, the richest income quintile had greater odds of visiting the dentist than the poorest income quintile, in line with the positive effect of income on oral healthcare utilisation [13]. A study among low income Canadians indicated oral healthcare utilisation as a competing financial demand for economically constrained adults [44]. In OECD countries, differences in visits between high and low-income groups were almost 20% (72% of wealthier individuals visited a dentist, compared with 54% among those from the lowest income quintile), indicating large socioeconomic disparities [28]. Low socioeconomic status may be associated with more emotional and physical consequences of seeking care when complications have occurred [45]. In Malaysia, health-related tax relief are available for complete medical examination and medical expenses for serious diseases for self, spouse, child, as well as medical expenses for parents but oral healthcare services are not considered for income tax relief [46]. Income tax relief for oral health examination for self, spouse, or child and oral healthcare expenses for parent(s) could be implemented in a similar way to that of medical healthcare as a way to promote utilisation of oral healthcare services in the country, especially in the private sector for those who can afford it, to reduce congestion in the public sector [4]. Andersen [22] noted that oral healthcare utilisation was more likely explained by predisposing social structure (e.g. education), predisposing health belief, and enabling factors (e.g. income).

The trend of oral healthcare utilisation among the population of all age groups in Malaysia has always been low. Recent studies indicated the prevalence of oral healthcare utilisation ranged from 22.4 to 23.7% between 2011 and 2019 [23, 32, 47]. Regular oral healthcare utilisation is important to prevent more complicated and costly procedures in the future. Therefore, it is important to improve the rate of oral healthcare utilisation among the population. Inequalities and inequities in oral healthcare services are prevalent worldwide [48–50]. Oral healthcare inequities in low and middle income countries are rooted in access to services and lack of preventative care [51]. Globally, lack of access to oral healthcare remains a major burden to public health. Although oral healthcare in Malaysia is accessible to all, there are still significant inequalities in oral healthcare utilisation, with the higher income population having better conditions to access these services [52]. Barriers in oral healthcare primarily centred on cost, public health prioritisation, and access; both organisational and geographical [53–56]. In Malaysia, studies have indicated dental fear, time constraints, dissatisfaction with the services rendered such as long waiting time and no immediate treatment given by the dentist, and perception of not having any oral health problems as barriers to oral healthcare utilisation [57, 58]. By understanding factors which influence utilisation of oral healthcare, improvement in oral healthcare utilisation could be achieved through appropriate interventions. Oral healthcare inequalities could be reduced through the implementation of effective and appropriate oral health promotion policies such as population-oriented preventive approach and integrated public health policies [59, 60].

Strengthening collaboration between the public and private sectors, non-governmental organisations (NGO), and social welfare groups to deliver oral healthcare outreach services to vulnerable groups such as the elderly could help improve oral healthcare access [4]. Enhancement of the existing domiciliary oral healthcare services for the elderly in Malaysia to cater to more individuals who are unable to attend oral healthcare clinics could also help boost utilisation [61]. Campaigns which advocate the importance of and interrelationship between general health and oral health through multi-sector collaboration and concerted efforts could help improve public awareness and health services utilisation [4]. In Malaysia, collaboration between the Ministry of Health Malaysia and various organisations not limiting to the Institute of Teacher Education, Oral Cancer Research and Coordinating Centre Malaysia and religious bodies were established to provide oral health talks and free oral examination through outreach programmes to the community they collaborate with [62]. Public–private collaboration such as the Alliance for a Cavity-Free Future Programme between the Ministry of Health Malaysia and the dental industry had a vision of achieving a future generation with zero cavity and better oral health [63]. Continuous monitoring and evaluation of existing programmes such as community empowerment programmes are essential for continuity of the programmes and their success in imparting oral healthcare knowledge to the public especially among those with lower levels of education [4]. In Malaysia, monitoring and evaluation of programmes are routinely done to gauge their effectiveness and acceptance, but in many instances there was poor uptake of the activities of the programmes by the community [27], which suggest a need to explore the public’s perception on the effectiveness of these approaches as well as barriers which led to the poor response of the programmes in future studies.

Improving the population’s social determinants of health could help improve oral healthcare access [59, 64]. In line with the universal health coverage agenda, oral health check-up and basic oral healthcare should be included as part of primary healthcare benefits package to strengthen healthcare financial mechanism [59]. In Malaysia, oral healthcare in the public sector is largely subsidised by the government and accessible to all citizens, yet utilisation is still low. The long waiting time to see the dentist in the public sector due to overcrowding in public facilities may have reduced the motivation of the population from their regular dental check-up [65]. In order to reduce this waiting time, the Ministry of Health Malaysia in year 2009 implemented a key performance indicator (KPI) for patient waiting time [65]. Over the years, Malaysia has seen an increase in the number of dentists in Malaysia, but without an appreciable increase in infrastructure or equipment [66] and this incompatibility between the number of professionals and facilities does not translate into better access to oral health for the public. Further study to understand the reasons for non-utilisation of oral healthcare despite the efforts poured by the Ministry of Health Malaysia to encourage participation is required so that appropriate interventions that cater to the needs of the population could be formulated to boost up oral healthcare utilisation in Malaysia.

This study used a large sample size involving nationwide population which enables generalisation of results across adults in Malaysia. One limitation to consider is that possible explanations for utilisation differences in oral healthcare like need factors (such as perception and attitudes to oral healthcare, and health literacy) [67], community-related factors (such as availability of health personnel and facilities, travelling cost, distance to facility, and waiting time) [68] and social determinants of health (such as social networks, social interactions, and culture) [69] were not captured in this study. Another limitation is that NHMS data is a twelve-month self-recall data which is subjected to potential recall bias and the possibility of under-reporting [70].

## Conclusion

Our study indicates that demographic and socioeconomic factors are strong determinants of oral healthcare utilisation in Malaysia with age, sex, marital status, education, income, and medical check-up in the last 12 months as the associated factors. Appropriate interventions to strengthen the existing programmes aimed to promote regular and timely oral health check-ups are needed to improve oral healthcare utilisation.

## Data Availability

To protect the privacy of the participants, the dataset that supports the findings of this article is not publicly available. The data may be requested from the corresponding author on reasonable request upon permission from the Director General of Health, Malaysia.

## Abbreviations

NHMS: National Health and Morbidity Survey
EBs: Enumeration Blocks
LQs: Living Quarters
SD: Secure Digital
NGO: Non-governmental Organisation
NCD: Non-communicable disease
CI: Confidence Interval
OR: Odds Ratio
OECD: Organisation for Economic Co-operation and Development
NOHSA: National Oral Health Survey of Adults
MREC: Medical Research and Ethics Committee
NMRR: National Medical Research Register
KPI: Key Performance Indicator
